# Laryngeal Squamous Cell Carcinoma Survival in the Context of Human Papillomavirus: A Systematic Review and Meta-analysis

**DOI:** 10.7759/cureus.2234

**Published:** 2018-02-26

**Authors:** Navid Ahmadi, Nima Ahmadi, Michael V Chan, Ya Ruth Huo, Niranjan Sritharan, Ronald Chin

**Affiliations:** 1 Faculty of Medicine, UNSW; 2 Colorectal Surgery, Royal Prince Alfred Hospital; 3 Department of Radiology, Concord Hospital; 4 ENT Department, Nepean hospital; 5 Department of Ent, University of Sydney

**Keywords:** human papilloma virus (hpv), scc (squamous cell cancer), larynx, laryngeal cancer, overall survival, survival outcome, oropharynx, head and neck cancer, orophahryngeal cancer

## Abstract

Background: Head and neck (H&N) squamous cell carcinoma (SCC) is a significant contributor to worldwide mortality and morbidity. Human papillomavirus (HPV) has been linked with H&N cancer and HPV-positive H&N SCC have been shown to have better survival outcomes.

Objective: To evaluate the effect of human papillomavirus (HPV) on laryngeal carcinoma (LSCC) survival outcomes and prognosis.

Method: A systematic review and meta-analysis were performed using the Preferred Reporting Items for Systematic Reviews and Meta-Analyses (PRISMA) guidelines. LSCC was confirmed based on histopathology, and HPV status was confirmed by either polymerase chain reaction, immunohistochemistry, and/or in-situ hybridization.

Results: There were 1214 studies which were identified, of which 14 studies were eligible for our review. A total of 2,578 cases of LSCC were included in analysis with 413 (16.0%) HPV-positive. Overall survival (OS) was not significant for HPV-positive LSCC in first five years (year one: OR 1.44 p=0.13; year two: OR 1.24 p=0.30; year three: OR 1.01 p=0.97; year four: OR 1.13 p=0.63; year five: OR 1.01 p=0.98). Disease-free survival (DFS) was similarly not significant for HPV-positive LSCC (year one: OR 1.08 p=0.68; year two: OR 1.22, p=0.31; year three: OR 1.13, p=0.69; year four: OR 0.93, p=0.80 and year five: OR 1.42, p=0.30). When studies are sub-divided into global regions, Chinese studies had better HPV-positive survival compared to North American studies in year five (OR 1.84 vs OR 0.46, p=0.04).

Conclusion: This is the first study of its kind to evaluate the survival impact of HPV-positive LSCC patients. Unlike oropharyngeal cancer, HPV status does not make a difference to OS or DFS in LSCC. This supports data that HPV is not a prognostic factor in squamous carcinoma of the larynx.

## Introduction

Squamous cell carcinoma of the head and neck is a significant contributor to worldwide morbidity and mortality. There are more than 500,000 new cases worldwide reported annually [1]. Laryngeal squamous cell carcinoma (LSCC) is the second most common head and neck malignancy worldwide with 151,000 new cases per year worldwide [2-3]. While there is a great amount of evidence that suggests alcohol and tobacco are causative agents in developing LSCC, there are increasing incidences of LSCC in non-smokers and non-drinkers [4-6].

There is great variation in reporting the incidence of HPV-positive LSCC in literature, however, a recent meta-analysis reports incidence at roughly 25% with minor variation depending on methods of testing [7]. While it is regarded that HPV-positive oropharyngeal squamous carcinoma has better survival outcomes, there is no evidence the same holds true for LSCC.

The aim of this study is to conduct a systematic review and meta-analysis to evaluate and quantify the effect of human papillomavirus (HPV) on survival outcome of LSCC and to assess regional variation across the globe.

## Materials and methods

Search strategy and selection criteria:

Using the Preferred Reporting Items for Systematic Reviews and Meta-analysis (PRISMA) guidelines, this study was conducted [8]. Electronic search of Medline, Embase, Cochrane Methodology Register, Cochrane Database of Systematic Reviews, Cochrane Central Register of Controlled Trials, Database of Abstracts of Reviews and Effectiveness, ACP Journal Club, and NHS Economic Evaluation Database was conducted from their respective conception to January 2017.

Utilising both MeSH terms and keywords, the search was conducted to ensure ideal sensitivity. The terms “larynx”, “squamous cell carcinoma” and “human papilloma virus (HPV)” along with their relevant variations were used. Relevant studies were identified by two independent reviewers (Na.A. and Ni.A.) and were extracted for further evaluations.

Inclusion criteria included histologically confirmed LSCC and HPV-DNA or p16 status was confirmed by polymerase chain reaction (PCR), immunohistochemistry (IHC), and/or in-situ hybridization (ISH). Studies were excluded if they did not provide overall survival (OS) or disease-free survival (DFS) comparing HPV-positive and HPV-negative LSCC. Search was limited to papers with minimum length of follow up of one year, English language, and human subjects. Abstracts, case-reports, conference presentation and any papers without original data were excluded.

Data extraction and critical appraisal:

Two independent investigators (Na.A. and Ni.A.) extracted data, with discrepancies between reviewers resolved by consensus. The senior investigators (N.S. and R.C.) reviewed final results.

Statistical analysis:

Meta-analysis was performed with Review Manager (Version 5.3. Copenhagen: The Nordic Cochrane Centre, The Cochrane Collaboration, 2014) using pooled OS and DFS of HPV-positive and HPV-negative LSCC [9]. Data was pooled using the DerSimionian and Laird’s random effect model at one, two, three, four, and five years post-diagnosis [10]. To assess publication bias, Egger’s linear regression test was utilized and heterogeneity was assessed and quantified using the I^2^ statistics to measure percentage variability among summary indices [11-12]. Statistical significance was set at 0.05.

## Results

**Figure 1:**
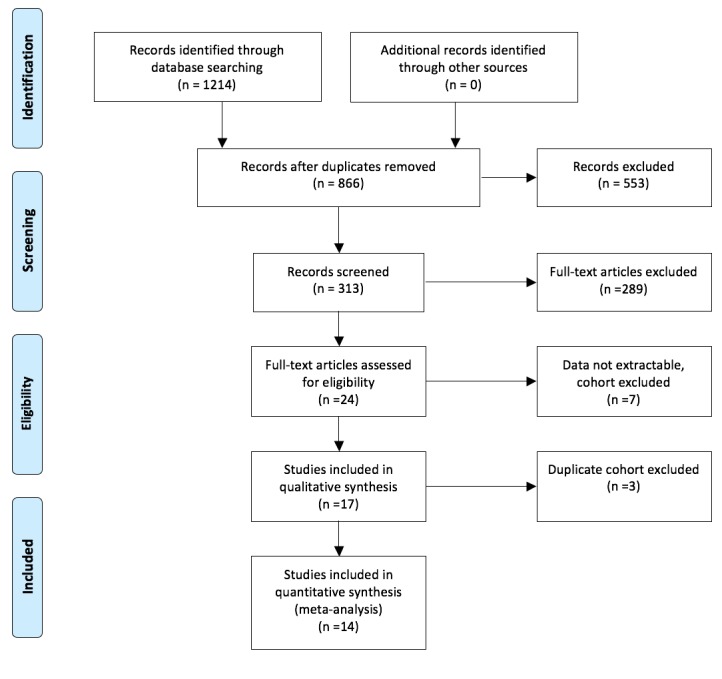
PRISMA Flow Diagram From: Moher D, Liberati A, Tetzlaff J, Altman DG, The PRISMA Group (2009). Preferred Reporting Items for Systematic Reviews and Meta-Analyses: The PRISMA Statement. PLoS Med 6(7): e1000097. doi:10.1371/journal.pmed1000097

Through the search of the nine databases, 1214 studies were identified. After excluding studies with duplicated cohorts, irrelevant studies, and studies with data not extractable, 14 studies were analyzed for statistical analysis. All the studies were case-control with two prospective studies, while the remaining 12 were retrospective. Bias was assessed using Egger’s test for publication bias and no publication bias was identified [11]. PRISMA guideline study selection is shown in Figure 1, with a description of studies in Table 1.

**Table 1:** Description of Studies Utilized in this Meta-analysis. NR – not recorded, ISH – In-situ hybridization, PCR – polymerase chain reaction, IHC – Immunohistochemistry, PE – paraffin embedded, FF – fresh frozen

Author	Year	Country	n	Study Period	Study Design	Prospective	Detection of HPV	Sample	p16	HPV DNA
Rivera-Pena	2016	USA (Puerto Rico)	83	1993-2005	Case-control	Retrospective	PCR	FF	NR	Y
Hernandez	2016	USA	101	1993-2004	Case-control	Retrospective	IHC	PE	Y	NR
Atighechi	2016	Iran	44	2007-2012	Case-control	Retrospective	PCR	PE	NR	Y
Young	2015	Australia	307	2002-2012	Case-control	Retrospective	IHC, ISH	PE	Y	Y
Wang	2015	USA	356	1995-2009	Case-control	Retrospective	PCR, IHC	PE	Y	Y
Larque	2015	Spain	45	NR	Case-control	Retrospective	PCR, IHC	FF, PE	Y	Y
Xu	2014	China	674	2006-NR	Case-control	Prospective	IHC	FF, PE	Y	Y
Lassen	2014	Denmark	479	1992-2012	Case-control	Retrospective	IHC	PE	Y	NR
Chung	2014	Multicentre	181	NR	Case-control	Prospective	IHC, ISH	PE	Y	NR
Jiang	2013	China	71	1997-2008	Case-control	Retrospective	IHC, ISH	PE	NR	NR
Stephen	2012	USA	79	NR	Case-control	Retrospective	PCR	PE	NR	Y
Duray	2010	Belgium	67	2001-2007	Case-control	Retrospective	PCR, IHC	PE	Y	Y
Morshed	2010	Poland	93	1999-2002	Case-control	Retrospective	PCR, IHC	PE	NR	Y
Clayman	1994	USA	78	Prior to 1989	Case-control	Retrospective	PCR	PE	NR	Y

Overall, 2578 LSCC cases were included in this analysis with 413 (16.0%) cases positive for HPV with 2051 (91.6%) male. HPV rates differed greatly between regions, as is summarised in Table 2. There was no difference noted in terms of OS of HPV-positive male (OR 0.63, 95% CI 0.20-1.92, p=0.41, I^2^=75%). Majority of LSCC were smokers (n=1252, 85.6%) and alcohol drinkers (n=703, 61.1%). Also, most of LSCC were well differentiated (61.2%) and only the minority were poorly differentiated (15.7%). In terms of tumor classification, there was an even spread between the grades (T1: 27.6%, T2: 22.1%, T3: 35.4, T4: 14.9%). Most of the LSCC had no regional node involvement (58.6%). Most patients were treated with the combination of radiotherapy and/or chemotherapy (42.4%), while 30.9% had surgery alone and 26.7% had surgery with chemotherapy and/or radiotherapy (Table 3).

**Table 2:** Breakdown of HPV Incidence Based on Region of the World HPV - Human Papillomavirus

Author	Year	n	HPV +	HPV -	
CHINA					
Jiang	2013	71	31 (43.7%)	40 (56.3%)	
Xu	2014	674	33 (4.9%)	641 (95.1%)	
Wang	2015	318	42 (13.2%)	276 ( 86.8%)	
	Total	1063	106 (10.0%)	957 (90.0%)	
EUROPE					
Morshed	2010	93	33 (35.5%)	60 (64.5%)	
Duray	2010	59	44 (74.6%)	15 (25.4%)	
Lassen	2014	479	65 (13.6%)	414 (86.4%)	
Larque	2015	45	4 (8.9%)	41 (91.1%)	
	Total	676	146 (21.6%)	530 (78.4%)	
NORTH AMERICA & CENTRAL AMERICA					
Clayman	1994	65	30 (46.2%)	35 (53.8%)	
Stephen	2012	77	21 (27.3%)	56 (72.7%)	
Hernandez	2016	101	8 (7.9%)	93 (92.1%)	
Rivera-Pena	2016	83	41 (49.4%)	42 (50.6%)	
	Total	326	100 (30.7%)	226 (69.3%)	
OTHERS					
Chung	2014	181	31 (17.1%)	150 (82.9%)	
Young	2015	288	19 (6.6%)	269 (93.4%)	
Atighechi	2016	44	11 (25.0%)	33 (75.0%)	
	Total	513	61 (11.9%)	452 (88.1%)	

**Table 3:** Demographics of studies involved in Meta-analysis NR – Not Recorded. N0 – no nodal metastasis N+  — nodal metastasis CRT — combination of chemotherapy and/or radiotherapy. *median age,

																			Differentiation		Nodal Metastasis		Location			Treatment			
	Author	Year		n		HPV +ve	HPV -ve	Mean Age		Age Range		Male (%)		Smoker (%)		Alcohol Drinker (%)		Well-Moderate		Poor	N0	N+	Supra-glottis	Glottis	Sub-glottis	Surgery alone		Surgery + CRT	CRT
	Rivera-Pena	2016		83		41	42	NR		NR		NR		NR		NR		NR		NR	NR	NR	NR	NR	NR	NR		NR	NR
	Hernandez	2016		101		8	93	NR		NR		NR		NR		NR		NR		NR	NR	NR	NR	NR	NR	NR		NR	NR
	Atighechi	2016		44		11	33	57.8		NR		90.9		NR		NR		NR		NR	NR	NR	NR	NR	NR	NR		NR	NR
	Young	2015		288		19	269	66*		36-88		93.9		88.2		NR		NR		NR	213	94	102	180	16	NR		95	207
	Wang	2015		318		42	276	59*		32-87		98.1		86.1		41.5		282		36	290	28	NR	NR	NR	228		90	NR
	Larque	2015		45		4	41	65		NR		100.0		NR		NR		17		28	NR	NR	13	12	16	NR		NR	NR
	Xu	2014		674		33	641	NR		NR		97.0		82.9%		65.0		NR		NR	363	311	172	461	31	320		354	NR
	Lassen	2014		479		65	414	NR		NR		81.0		NR		NR		NR		NR	156	232	NR	NR	NR	NR		NR	NR
	Chung	2014		181		31	150	NR		NR		NR		NR		NR		NR		NR	NR	NR	126	50	5	NR		NR	NR
	Jiang	2013		71		31	40	61.6		NR		97.2		NR		NR		60		11	50	21	32	39	NR	32		39	NR
	Stephen	2012		77		21	56	NR		NR		75.3		94.8		97.0		61		15	NR	NR	28	28	1	26		NR	51
	Duray	2010		59		44	15	NR		NR		97.0		NR		NR		66		1	56	11	15	35	NR	67		NR	NR
	Morshed	2010		93		33	60	NR		NR		83.9		87.9		74.7		79		14	49	44	54	35	4	NR		NR	NR
	Clayman	1994		65		30	35	60		NR		83.1		NR		NR		NR		NR	NR	NR	NR	NR	NR	22		23	23
	Total			2578		413 (16.0%)	2165 (84.0%)	-		-		91.6		85.6		61.1		565 (84.3%)		105 (15.7%)	1177 (61.4%)	741 (38.6%)	542 (37.3%)	840 (57.7%)	73 (5.0%)	695 (44.1%)		601 (38.1%)	281 (17.8%)

**Figure 2:**
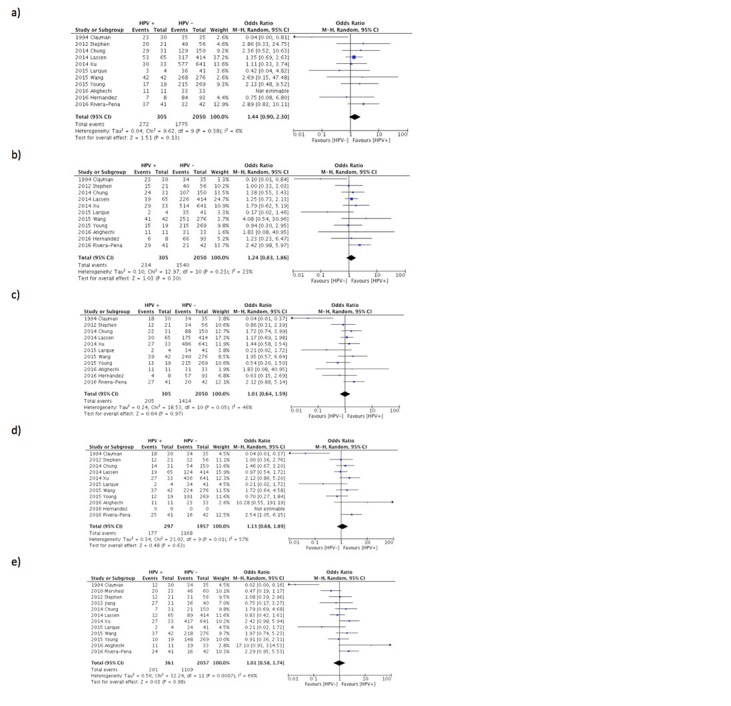
Forest plot comparing overall survival (OS) comparing HPV-positive LSCC to HPV-negative LSCC a - year 1, b - year 2, c - year 3, d - year 4, e - year 5 LSCC - Laryngeal Squamous Cell Carcinoma

**Figure 3:**
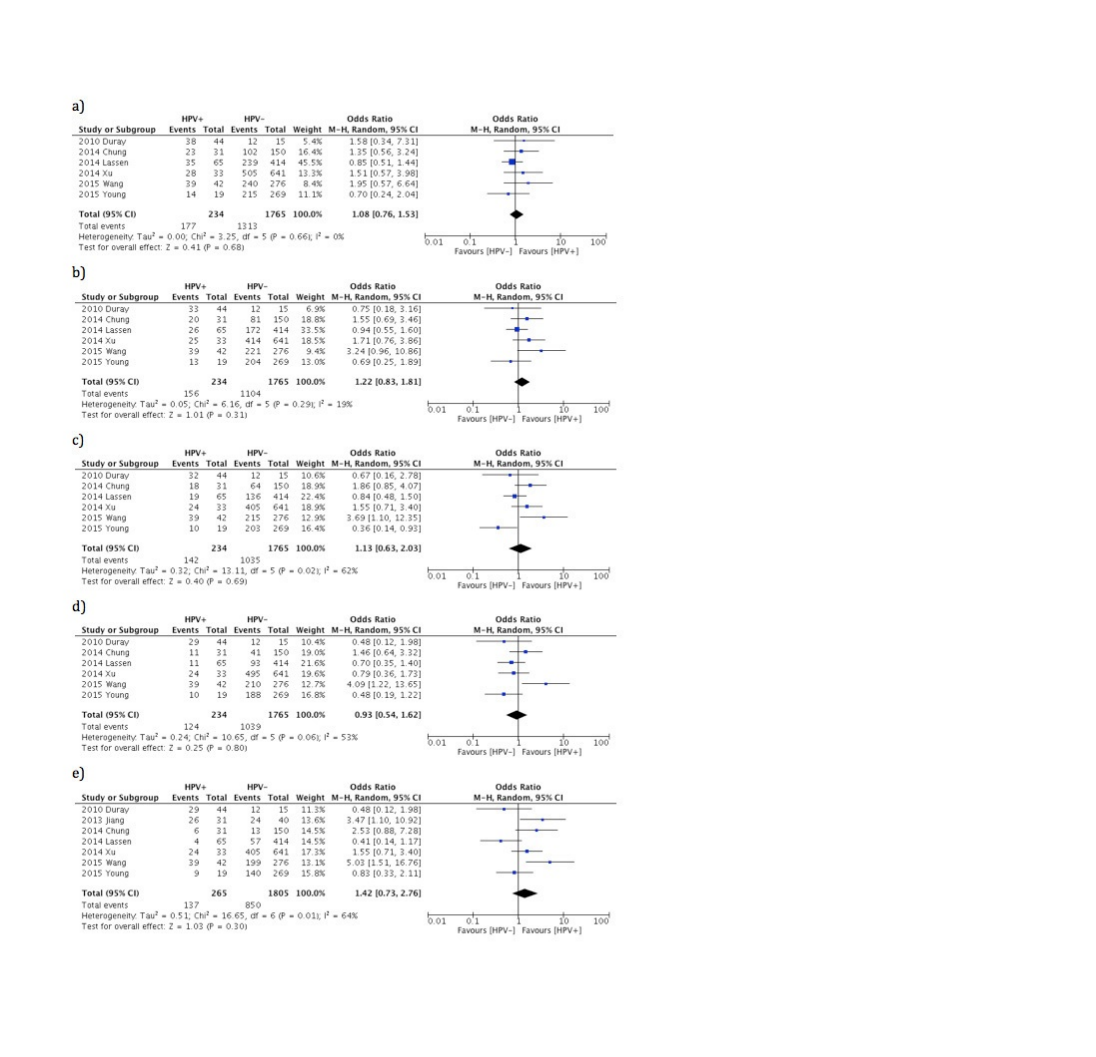
Forest plot comparing disease-free survival (DFS) comparing HPV-positive LSCC to HPV-negative LSCC a - year 1, b - year 2, c - year 3, d - year 4, e - year 5 LSCC - Laryngeal Squamous Cell Carcinoma

In terms of survival, there was no difference noted in terms of OS in the first five years post-diagnosis (Figure 2). However, from year three to five post-diagnosis, there was a significant heterogeneity noted between studies (year three: I^2^=46%, year four: I^2^=57%, year five: I^2^=66%). Similarly, there is no difference noted in DFS in the first five years post-diagnosis. There is also significant heterogeneity in third- and fifth-year post-diagnosis (year 3: I^2^=62%, year 5: I^2^=64%). Similarly, there was no significance between DFS in the context of HPV (Figure 3).

When OS is stratified based on location, studies from China show better OS of HPV-positive LSCC in fourth and fifth-year post-diagnosis (OR=1.92, p=0.05, and OR=1.84, p=0.05 respectively) compared to North America in the same year (OR=0.63, p=0.39, and OR=0.46, p=0.48). This demonstrates a significant difference noted between the regions in fifth-year post-diagnosis (p=0.04). Looking at heterogeneity within regions, there is a significant heterogeneity noted in third- to fifth-year post-diagnosis (p=0.05, p=0.01, and p<0.01 respectively). The results are summarised in Table 4.

**Table 4:** Breakdown of odds ratio (OR) of HPV-positive LSCC as compared to HPV-negative LSCC subdivided based on global regions HPV - Human Papillomavirus LSCC - Laryngeal Squamous Cell Carcinoma

Regions	No. of studies	OR	95%CI	p-value	I^2^	Hetrogeneity (p-value)
Year 1						
China	2	1.27	0.41-3.88	0.68	0%	0.57
Europe	2	1.25	0.65-2.37	0.50	0%	0.36
N. America & C. America	4	0.97	0.18-5.18	0.97	63%	0.04*
Others	3	2.24	0.78-6.48	0.14	0%	0.93
Between Subgroups				0.78	6%	0.38
Year 2						
China	2	2.14	0.83-5.49	0.11	0%	0.47
Europe	2	0.61	0.09-3.96	0.61	68%	0.08
N. America & C. America	4	0.96	0.32-2.87	0.95	61%	0.05*
Others	3	1.21	0.61-2.43	0.59	0%	0.85
Between Subgroups				0.57	23%	0.23
Year 3						
China	2	1.60	0.77-3.31	0.21	0%	0.69
Europe	2	0.67	0.14-3.29	0.63	59%	0.12
N. America & C. America	4	0.62	0.17-2.20	0.46	75%	<0.01
Others	3	1.07	0.44-2.57	0.89	35%	0.22
Between Subgroups				0.54	46%	0.05*
Year 4						
China	2	1.92	0.99-3.73	0.05*	0%	0.76
Europe	2	0.63	0.16-2.44	0.51	47%	0.17
N. America & C. America	4	0.63	0.10-3.78	0.61	85%	<0.01*
Others	3	1.28	0.68-1.89	0.60	45%	0.16
Between Subgroups				0.39	57%	0.01*
Year 5						
China	3	1.84	1.00-3.36	0.05*	0%	0.41
Europe	3	0.62	0.36-1.08	0.09	5%	0.35
N. America & C. America	3	0.46	0.06-3.86	0.48	89%	<0.01*
Others	3	1.70	0.59-4.93	0.33	52%	0.13
Between Subgroups				0.04*	66%	<0.01*

## Discussion

The pathogenesis of HPV and its effect on patient treatment and survival outcomes is an area of great importance in head and neck oncology. This study evaluates the current literature with regards to the pathogenesis of HPV as it relates specifically to laryngeal cancer and suggests that unlike in oropharyngeal cancer, HPV in the larynx has little to no effect on survival outcome.

HPV consists of double-stranded DNA genomes with more than 100 genotypes, of which more than 24 genotypes are detected in head and neck cancers [13-17]. HPV produces the malignant transformation of cells by producing E6 and E7 proteins that act together to lock cells in a forward replication phase [18]. As a result, HPV-positive oropharyngeal SCC (OSCC) is different to their HPV-negative counterparts, in that they have different patterns of dissemination and lower incident of metachronous primary tumors [19]. This may explain why HPV-positive OSCC have better responses to chemotherapy and radiotherapy, such that the latest American Joint Committee on Cancer (AJCC) divided staging for OSCC into different categories based in HPV status in the eight edition [20].

HPV has a propensity for tumorigenesis in the tonsillar crypts, which are deep recesses with a discontinuous basement membrane, and vasculature, which may explain the early propensity for lymphovascular invasion and early regional nodal spread [21-22]. This contrasts with laryngeal epithelium which is squamous epithelium with less abundant lymphatics particularly in the glottis [21-22].

While there is a large body of literature looking at HPV in oropharyngeal cancer, little research has focused on laryngeal pathology with regards to HPV. In this study, we demonstrate that there is no difference in survival in the context of OS and DFS based on HPV status. This suggests that HPV is not a significant driver of LSCC, and as such HPV status has limited clinical relevance in LSCC.

Recent meta-analysis by Ndiaye and colleagues showed HPV prevalence of LSCC is 22.1% (95% CI 16.4-28.3) while another meta-analysis demonstrated that the prevalence was 26.8% or 20.3% depending on whether PCR-testing for HPV was utilized or not [6-7]. This is lower than the incidence of HPV (45.8%; 95% CI 38.9–52.9%) in OSCC in the same study [6]. This demonstrates selective uptake of HPV in oropharynx as compared to larynx, and further suggests that HPV is likely not a strong indicator of LSCC. The prevalence of LSCC in this study (16%) is lower than the two above-mentioned meta-analyses. This may represent an inherent selection bias, as only studies with survival outcome and HPV status were included. As a result, the external validity of the current literature needs to be questioned in terms of providing accurate survival data on LSCC in context of HPV.

Another explanation for the different prevalence of HPV is the heterogeneity between different regional populations. The prevalence HPV-positive LSCC of Chinese cohort is 10%, while the prevalence of the rest of the world is 20.3%. While others have shown similar heterogeneity, their reported prevalence is different to our study. Ndiaye, et al. (2014) show the prevalence of HPV is 28.3% in Asia and 18.8% in North America, while Gama, et al. (2016) show point estimate of 0.246 (95%CI: 0.201-0.298) in North America to 0.399 (95%CI: 0.323-0.479) [6-7]. The lower rates in the Chinese population may, to some extent, account for a higher smoking rate in China, compared to North America.

There was no difference noted between HPV-positive and HPV-negative LSCC in terms OS and DFS. However, there was significant heterogeneity noted in studies. As mentioned previously, the AJCC now considers HPV-positive and HPV-negative OSCC different disease entities [20]. This is because HPV-positive OSCC also has better survival outcomes compared to HPV-negative OSCC [23-25]. However, the same does not seem to follow for LSCC. The difference in HPV prevalence between oropharynx and larynx is still not completely well understood and it is uncertain why HPV affects OSCC and LSCC differently [26].

There is significant regional variation in LSCC survival. This is most pronounced in the fifth year post-diagnosis, where there is a significant difference between the stratified cohorts (Table 4). As mentioned above, Xu, et al. attribute the low prevalence of HPV in Chinese cohorts to high rates of smoking within China which may explain the variability in terms of prevalence and survival of LSCC patients in Chinese cohort [27]. Furthermore, the variability also raises the concern regarding the external validity of various studies performed across the many centers around the world and calls for investigation of the cause of this difference. More molecular and epidemiologic studies are needed to evaluate the relationship of HPV and laryngeal cancers [26].

Limitations

This meta-analysis is limited by the number of studies with adequate data to allow for sub-analysis of outcomes and further multivariate analysis. There were also a limited number of studies from various regions of the world, with North American and Chinese studies accounting for most of the cohort of our meta-analysis. Further research is required to better assess survival outcome of LSCC based on HPV status.

## Conclusions

This is the first meta-analysis to compare HPV-positive LSCC with smoking and alcohol-related LSCC and compare survival outcomes. This is the largest laryngeal meta-analysis looking at survival outcomes in laryngeal SCC with 2578 cases. Even though the study showed heterogeneity across regions in the world, particularly North America and China, the study showed that unlike oropharyngeal squamous cell carcinoma, HPV-positive LSCC does not have a better survival outcome compared to smoking and alcohol-related LSCC.
